# Efficacy of an Ayurvedic Intervention as an Adjunct to Standard Care in Preventing Acute Pain Crises in Sickle Cell Anemia: Protocol for a Randomized Controlled Trial

**DOI:** 10.2196/76576

**Published:** 2025-12-31

**Authors:** U R Sekhar Namburi, Priya Thakare, Vaishali Kuchewar, Manish Deshmukh, Kalpana Kachare, Madhukar Tikas, Deepa Makhija, Bhogavalli Chandra Sekhara Rao, MN Suryawanshi, Satish Mahajan, Abha Sharma, N Srikanth, Ravinarayana Acharya

**Affiliations:** 1 Regional Ayurveda Research Institute, Nandanvan, Nagpur, 440024, India, 91 8055595355; 2 Mahatma Gandhi Ayurved College Hospital and Research centre, Wardha, India; 3 Datta Meghe Institute of Higher Education & Research (Deemed to be University), Wardha, India; 4 Central Council for Research in Ayurvedic Science, New Delhi, India; 5 Datta Meghe Medical College, Wanadongari, Nagpur, India; 6 Datta Meghe Institute of Medical Sciences, Wardha, India

**Keywords:** hemoglobin, raktavaha srotas dushti, dadimadi ghrita, Ayush-RP, pandu

## Abstract

**Background:**

Sickle cell anemia (SCA) represents a major health concern among the tribal population of India, with frequent acute pain crises significantly compromising the quality of life of the affected individuals. As an inherited disorder, there is no definitive cure for the condition. Hydroxyurea remains the primary therapeutic option and is typically used for lifelong management, although it may be associated with certain side effects. In light of the pressing need for a safe, accessible, and effective alternative for long-term care, this study is planned to explore the potential of Ayurveda in managing pain crises alongside conventional standard care.

**Objective:**

This study aims to evaluate the efficacy of Ayurvedic intervention in preventing acute pain crises in individuals with SCA and improving their quality of life.

**Methods:**

This study is designed as a randomized, active-controlled, open-label clinical trial. Participants diagnosed with SCA are enrolled in the study according to the selection criteria. The intervention group receives Ayurvedic interventions, namely *dadimadi ghrita* and Ayush-RP, along with standard care, whereas the control group receives standard care only. The intervention is administered over a period of 8 months. Participants are evaluated on the 30th, 60th, 105th, 150th, 195th, and 240th days to assess changes in the frequency of pain crises and quality of life.

**Results:**

This study was initiated on September 5, 2023. Total enrollment of 1510 participants has been completed by screening 1644 participants. As of August 14, 2025, a total of 1137 participants have successfully completed the study, 280 (24.62%) are continuing, and 93 (8.18%) have dropped out.

**Conclusions:**

This study aims to establish the efficacy and safety of Ayurvedic interventions as part of an integrated approach to managing SCA, with a focus on reducing the frequency of pain crises and improving patients’ overall quality of life.

## Introduction

Sickle cell anemia (SCA) is a hereditary condition within the broader group of sickle cell diseases (SCDs). It is one of the most prevalent monogenic disorders worldwide and is particularly common among the tribal populations in India [1,2]. SCA is a disease caused by a mutation in the β-globin gene, leading to the production of abnormal hemoglobin, which binds with other abnormal hemoglobin molecules within the red blood cells (RBCs) to cause rigid deformation of the cell. The abnormally shaped RBCs are unable to pass through the small vascular channels, resulting in sludging and congestion within the vascular beds, tissue ischemia, and infarction. The frequent occurrence of infarction in individuals with SCA is a principal cause of the early clinical manifestation, the acute pain crisis, often ascribed to bone marrow infarction [3]. The vaso-occlusive pain episodes frequently affect the limbs; abdomen; ribs; sternum; spine; and, occasionally, the skull, often elicited by illness and accompanied by clinical features such as fatigue, loss of appetite, anemia, jaundice, pneumonia, and recurrent infections. The life expectancy for those with SCA ranges from 42 to 48 years, with 50% of affected children dying before the age of 5 years, primarily due to recurring infections [4].

According to a survey by the Indian Council of Medical Research, the sickle cell gene is present in 5% to 34% of India’s various tribal groups due to genetic predisposition [5]. Maharashtra is one of the states in India with a high prevalence of SCA, where the Vidarbha region exhibits a significantly higher prevalence, with 62% of the total cases concentrated in the Nagpur division [6].

While there is no cure for SCA, the quality of life of affected individuals can be improved through adequate management of the disease. Hydroxyurea, a ribonucleotide reductase inhibitor, is currently considered the standard treatment in the management of SCD. It has demonstrated a reduction in painful episodes, acute chest syndrome, and mortality [7]. Folic acid is given as a prophylactic drug in all patients [8]. Blood transfusion also plays a vital role in managing severe complications such as acute chest syndrome, heart failure, stroke, and aplastic crises [9]. In SCA, acute pain typically requires management with opioids and hydration. However, the conventional treatment also has certain side effects. Hydroxyurea is an extremely toxic drug [7] and requires intensive monitoring to prevent side effects such as neutropenia, bone marrow suppression, elevation of hepatic enzymes, anorexia, nausea, vomiting, and infertility [10]. Blood transfusion carries risks such as iron overload and viral transmission, necessitating careful and continuous monitoring [9]. Effective management of pain is also challenging due to difficulty in accurately identifying its underlying cause and concern about opioid addiction [11]. Furthermore, the disease requires lifelong therapy and imposes a significant macroeconomic burden due to severe complications. Hence, there is a need for evaluating newer alternatives for effective and safe long-term management of SCA.

In Ayurveda, hereditary diseases are classified as *adibalapravritta vyadhi*, which refers to disorders originating from the impairment of the sperm or ovum. These conditions are attributed to genetic imbalances inherited from both parents and are considered *asadhya* (incurable). However, integrating the principles of Ayurveda with modern medicine can help reduce the frequency of painful crises and improve the quality of life of the patients. Formulations that enhance the function of the hematopoietic system (*raktavaha srotasa*) and boost the body’s vitality and strength may be beneficial in managing the condition [12]. Therefore, this study is planned with an aim to explore other alternatives for reducing pain crises associated with SCA, thereby enhancing the quality of life for affected patients. In a pilot study, Ayush-RP, a coded formulation developed for the management of SCA, demonstrated significant improvements in pain crises and quality of life for participants [13]. *Dadimadi ghrita* is a classical formulation used in the management of *pandu roga* (anemia) [14]. Furthermore, it mitigates the pain and increases the overall strength of the body [15,16]. Hence, both formulations were selected for this study. The primary objective of this study is to evaluate the efficacy of Ayurvedic interventions as an add-on to standard care for the prevention of acute pain crises in SCA. Secondary objectives include evaluating the safety of the Ayurvedic interventions, assessing their effects on various pathological and biochemical parameters, and measuring the quality of life of participants with SCA.

## Methods

### Study Design

This is a randomized, active-controlled, open-label clinical trial.

### Study Setting

Participants are screened at 3 centers: Civil Hospital in the Gadchiroli district; Acharya Vinoba Bhave Rural Hospital, Datta Meghe Institute of Higher Education and Research (DMIHER), Sawangi (Meghe), Wardha district; and Shalinitai Meghe Hospital and Research Centre in Wanadongri, Nagpur, Maharashtra, India. However, enrollment and assessment are conducted only at the latter 2 centers.

### Study Participants

#### Inclusion Criteria

Participants aged 18 to 50 years of any gender diagnosed with SCA confirmed via hemoglobin electrophoresis showing the HbSS pattern are eligible for this study. They must meet the following laboratory criteria: hemoglobin of >6%, platelets of >100,000 per cubic millimeter, serum creatinine of ≤1.5 mg/dL, international normalized ratio of ≤2.0, and partial thromboplastin time of ≤48 seconds. Additionally, participants should be willing to adhere to the study procedures and follow-up schedule and provide written informed consent.

#### Exclusion Criteria

Individuals are excluded from the study if they have a history of 5 or more hospitalizations for pain in the previous 12 months; require transfusions more frequently than once every 3 months; have a history of bleeding diathesis or a clinically overt stroke within the previous 2 years; are receiving chronic anticoagulation or antiplatelet therapy; have previously undergone a hematopoietic stem cell or solid organ transplant or recent RBC transfusion (within 60 days); have a history of cardiac arrhythmia, acute coronary syndrome, myocardial infarction, or stroke; or have symptomatic congestive heart failure. Individuals with chronic liver disease, alanine transaminase or aspartate transaminase levels exceeding 3 times the upper limit of normal, hypertension (blood pressure of ≥160/100 mm Hg), or clinically significant infections are also excluded. Furthermore, those unable to take oral medications or with malabsorption syndrome, active substance abuse, renal dysfunction (serum creatinine of >1.5 mg/dL for male individuals or >1.1 mg/dL for female individuals), uncontrolled pulmonary dysfunction (asthma or chronic obstructive pulmonary disease), or other severe comorbidities are excluded. Individuals with clinical manifestations such as hepatitis, glycated hemoglobin of ≥8%, AIDS or HIV, malignancy, or hyperthyroidism or currently participating in another clinical trial are also ineligible. Women who are planning conception, pregnant, or lactating are excluded, as well as individuals with conditions that the principal investigator deems may pose a risk to study safety or integrity. In addition, those with heterozygous sickle cell trait (AS pattern) or a history of severe sickle cell crises are excluded.

#### Rationale for Selection Criteria

The age group of 18 to 50 years was selected for this study as the incidence of SCA beyond the age of 50 years is low and the presence of age-related comorbidities in older individuals may introduce variability and potential confounding factors. Additionally, the previously reported maximum life expectancy for individuals with SCA is approximately 47 years [17]. Patients below the age of 18 years were not included in this study as they are considered a vulnerable population.

#### Withdrawal Criteria

Withdrawal from the study may occur if the participants choose to leave, if their condition deteriorates, or if they develop any health conditions listed in the exclusion criteria during the study. Participants who withdraw undergo a final examination if possible. In cases in which a participant leaves against medical advice, a final telephone interview is conducted to assess their health status. The reasons for withdrawal are documented in the case report forms (CRFs).

### Study Intervention

Participants in both groups receive standard care, which includes folic acid tablets of 400 mcg once daily after meals and hydroxyurea tablets of 500 mg once daily after meals in accordance with operational guidelines for the management of SCA [18]. In addition to standard care, participants in the intervention group are administered *dadimadi ghrita* [19] at a dosage of 12 grams twice daily before meals with lukewarm water and a tablet of Ayush-RP at a dosage of 500 mg twice daily after meals with lukewarm water. In both groups, each medication is administered orally for a duration of 8 months.

The interventional drug *dadimadi ghrita* is procured from a good manufacturing practices–certified manufacturer—Indian Medicines Pharmaceutical Corporation Limited. The other interventional drug, Ayush-RP, is procured from the Ayurveda pharmacy of the Central Ayurveda Research Institute, Jhansi, Uttar Pradesh, India. Both interventional drugs are prepared in accordance with the standards outlined in the Ayurvedic Formulary of India. The quality and safety parameters of *dadimadi ghrita* have been validated as per Ayurvedic Pharmacopoeia of India standards.

### Drug Compliance

Drug compliance forms are issued to the enrolled participants on each visit. Participants with a compliance of 80% or more continue in the study. Compliance is assessed at every visit by evaluating the approximate quantity of the interventional drugs consumed, confirmed with the quantity remaining in returned containers.

### Outcome Measures

The primary outcome of the study is a change in the incidence or frequency of sickle cell–related pain crises.

Secondary outcomes include changes in complete blood count, hemoglobin levels, hemoglobin variants (HbS, HbF, and HbA), lactate dehydrogenase, indirect bilirubin, free haptoglobin, potassium, and the C-reactive protein inflammatory marker. Secondary outcomes will also assess changes in the intensity of pain (measured via the visual analog scale), frequency of bleeding episodes, frequency of blood transfusions, hospital admissions and duration of stay, the incidence and severity of adverse events (AEs), and analgesic or opioid use. Changes in fatigue, measured using the Modified Fatigue Impact Scale, and quality of life, assessed using the 12-Item Short Form Health Survey, will also be evaluated in this study.

### Safety Outcomes

The safety of the study intervention is evaluated by recording the incidence of AEs, serious AEs, or adverse drug reactions on every scheduled visit. All AEs during the study timeline, if they occur, are recorded and monitored as per the good clinical practice guidelines. The safety assessment is conducted through hematological and biochemical tests, as mentioned previously.

### Duration and Timeline of the Study: 36 Months

The preparatory period comprises 6 months for engagement of project staff, finalization of CRFs, procurement of interventional drugs and insurance, preparation of the investigators’ brochure, and investigator training, among other aspects. The enrollment period comprises 16 months, and assessment of participants will be conducted on the 30th, 60th, 105th, 150th, 195th, and 240th days. The intervention will last for 8 months. Statistical analysis and preparation of the technical report and manuscript will last for 6 months.

### Study Procedure

#### Overview

**Figure 1.**
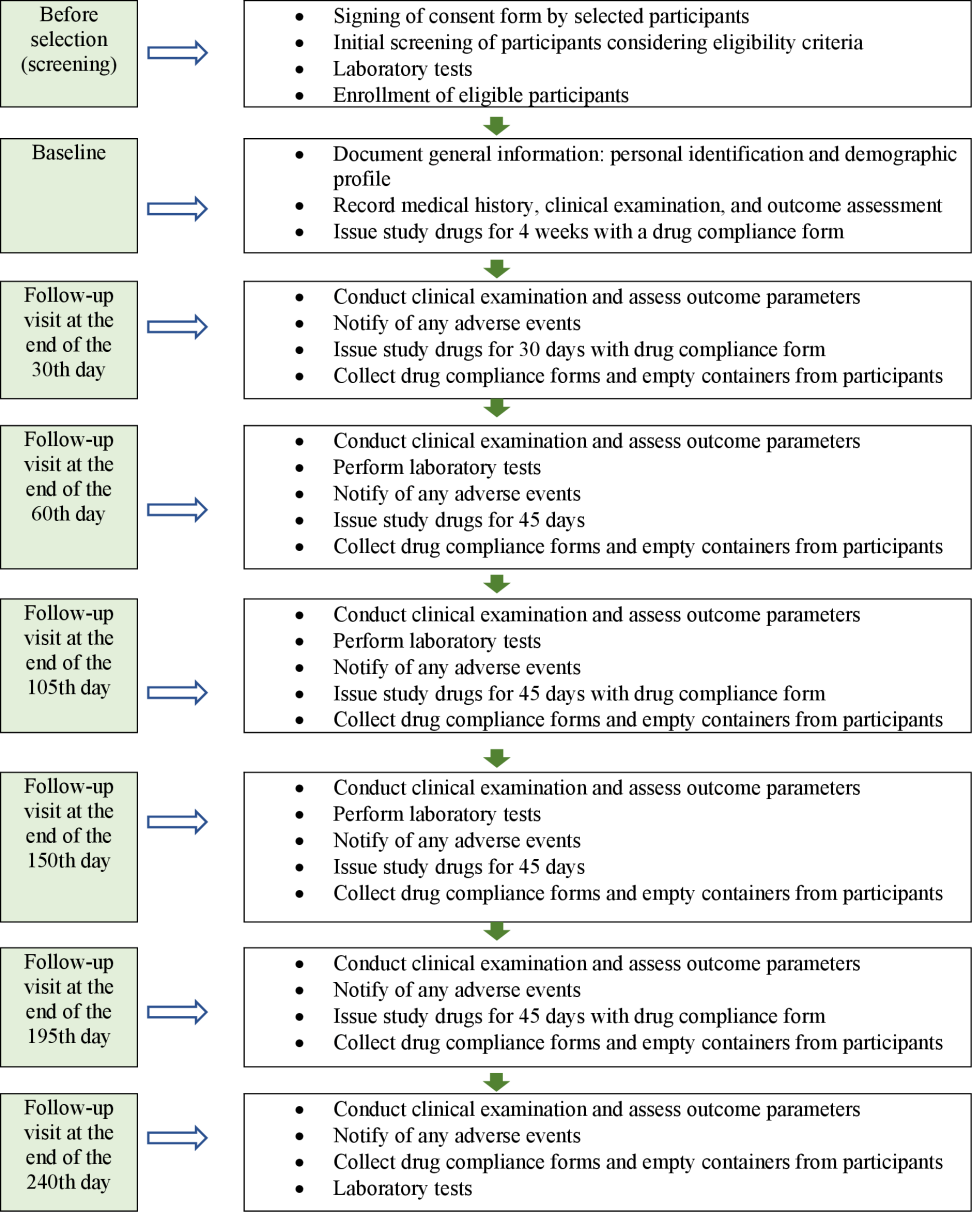
Study flow diagram.

The participants diagnosed with SCA are enrolled based on the selection criteria from the respective outpatient departments of the centers. To ensure adequate participant enrollment and achieve the target sample size, patients visiting the outpatient departments of hospitals at each site will be screened. Additionally, data will be collected through nongovernmental organizations, government hospitals, and specially organized camps. Participants are evaluated for demographic details and medical history at baseline and are provided with the study drugs at each subsequent visit (30th, 60th, 105th, 150th, 195th, and 240th days). During the visits, participants are assessed for primary and secondary outcomes. The hematological tests, namely complete blood count, erythrocyte sedimentation rate, bleeding time, clotting time, prothrombin time or international normalized ratio, free haptoglobin, peripheral smear, and complete electrophoresis, along with the biochemical tests, namely fasting blood sugar levels, glycated hemoglobin, liver function test, renal function test, C-reactive protein, and routine urine examination, are conducted at baseline and on the 60th, 150th, and 240th days. Radiological tests (electrocardiogram and ultrasonography) are conducted at baseline and the 240th day. For each visit, a window of 7 days from the scheduled date is allowed to minimize dropouts due to unavoidable circumstances. The participants are instructed to immediately inform if they encounter any adverse reaction or event throughout the study (Figure 1 and Table 1).

**Table 1.** Study schedule.

	Screening	Baseline	End of the 30th day	End of the 60th day	End of the 105th day	End of the 150th day	End of the 195th day	End of the 240th day
Informed consent	✔							
Demographics and medical history	✔	✔	✔	✔	✔	✔	✔	✔
Laboratory tests	✔			✔		✔		✔
Clinical examination	✔	✔	✔	✔	✔	✔	✔	✔
Assessment of ADRs^a^			✔	✔	✔	✔	✔	✔
Assessment of drug compliance			✔	✔	✔	✔	✔	✔
Issue of trial drugs at every visit		✔	✔	✔	✔	✔	✔	

a ADR: adverse drug reaction.

#### Participant Selection and Blood Sample Collection

Blood samples will be collected by the participating centers as follows:

- For the Wardha division, samples will be obtained from the Sickle Cell Unit, Acharya Vinoba Bhave Rural Hospital, DMIHER, Wardha.
- For the Gadchiroli division, samples will be collected from patients identified in the list provided by the Gadchiroli district health office.
- Similarly, for the Nagpur division, patients listed by the Nagpur district health office will serve as the reference for sample collection.

All blood samples collected from these divisions will be transported to the Central Research Laboratory at DMIHER, Wardha, following the prescribed guidelines.

#### Blood Sampling Schedule

Participants’ blood samples will be collected at 4 time points: during screening and subsequently on the 60th, 150th, and 240th days.

### Sample Size

Considering the primary outcome of this study as change in the incidence of sickle cell–related pain crises, the sample size is 1510, with 755 (50%) participants in each group (Textbox 1).

Textbox 1. **Justification for sample size calculation.**

- Level of significance (α): .05
- Z1-α (type 1 error): 1.64
- Power of test (1 – β): 0.80
- Z1-β (type 2 error): 0.84
- P1 (Expected proportion in control group): 35.4% (0.354)
- True proportion difference between the 2 groups in the sickle cell population (ε): 5% (0.05)
- P2 (Expected proportion in experimental group): 40.4% (0.404; assumed)
- Clinically acceptable for superiority (δ) single tail: 11.5% (0.115; expected)
- Sample size: 1367
- Dropout rate: 10%
- Total sample size: approximately 1504 to 1510
- Final sample size for each group: sample size calculation for superiority (parallel design) using the proportion difference when the end point is qualitative via the formula, $n=\frac{2\times (Z_{1-\alpha }+Z_{1-\beta }{)}^{2}\left(P_{1}(1-P_{1})+P_{2}(1-P_{2})\right)}{(\varepsilon -\delta {)}^{2}}$

### Randomization and Allocation Concealment

Participants are randomly assigned to either the intervention or control group at a ratio of 1:1 according to a prespecified randomization scheme prepared using a computerized system. To prevent selection bias, the randomization sequence is concealed using the sequentially numbered opaque sealed envelope method. Following the baseline assessment, an envelope with the slip containing the details of the allocated group is opened by the participants in front of the principal co-investigator. The participants are then assigned to the designated groups.

### Blinding

This study was conducted as an open-label trial, and no blinding was implemented. As preparing a placebo for one of the interventions, *dadimadi ghrita*, was not feasible, blinding was not planned.

### Data Collection

All data related to this study are being properly documented in both the CRFs and electronic format. Investigators and project staff at the participating centers have undergone training to ensure standardized data collection procedures. All source documents supporting the entries in the CRFs are kept readily accessible. These documents are securely stored at the study centers. The records and data collected during the study are stored in password-protected folders on secure computers and external storage devices, with access restricted only to the investigator.

### Concomitant and Rescue Medication

All participants are properly counseled not to take any medications other than the study drugs without prior consultation with the investigators. Concomitant therapy is continued for conditions such as diabetes mellitus, hypertension, or any other illnesses that are not listed in the exclusion criteria. Investigators may prescribe additional medications as needed to ensure reasonable supportive care during the study period. Details of all such interventions are recorded in each participant’s CRF. Any administration of rescue medication in the event of a medical emergency is also documented in the CRF. Participants are advised to report any new symptoms, unusual occurrences, or health concerns to the investigator immediately.

### Statistical Analysis Plan

The statistical analysis will be conducted after data collection, with a verification of their accuracy and limitations. Categorical variables will be presented as numbers and percentages, and pre- and posttrial comparisons will be analyzed using the McNemar chi-square test or Cochran *Q* test. Continuous variables such as scores and laboratory parameters will be assessed using the paired or unpaired 2-tailed *t* test if the data follow a normal distribution or the Wilcoxon test if the data are nonnormally distributed. For analysis of assessment parameters across multiple follow-ups, the repeated measures will be analyzed using ANOVA for normally distributed data or the Friedman test or Cochran *Q* test for nonnormally distributed or categorical data. Data with a normal distribution will be reported as means and SDs, and nonnormally distributed data will be presented as medians with IQRs. A 5% level of significance (*P*<.05) will be applied throughout the analysis, and the SPSS software (version 29.0; IBM Corp) will be used to conduct all statistical tests.

### Data Monitoring

The committee responsible for monitoring the study systematically evaluates its progress through scheduled review meetings and site visits as necessary to ensure rigorous adherence to the study protocol, as well as the completeness, accuracy, and consistency of the data. Additionally, the committee ensures compliance with relevant local regulations pertaining to the conduct of clinical research. An interim analysis will be conducted upon completion of the intervention period for at least 25% of the participants.

### Study Drug Management and Accountability

The study drugs are securely stored under the supervision of the investigators. Detailed records of the dispensed study drugs are maintained to prevent any discrepancies between the quantity dispensed and the amount remaining or returned.

### Ethical Considerations

#### Overview

This study is being conducted in accordance with the ethical principles outlined in the Declaration of Helsinki for biomedical research and the Indian Council of Medical Research Ethical Guidelines for Biomedical Research on Human Participants (2006), which are consistent with the International Council for Harmonisation of Technical Requirements for Pharmaceuticals for Human Use guideline for good clinical practice [20]. Recruitment started after obtaining approval from the institutional ethics committee and registration in the Clinical Trials Registry–India (Ctri/2023/04/052141).

#### Protocol Amendment

Any deviations from the study protocol will be implemented after obtaining prior approval from the institutional ethics committee.

#### Informed Consent

Written informed consent is obtained from all participants, with the benefits and limitations of the study clearly outlined in the consent forms and explained to participants by the investigators or senior research fellow before the commencement of the study.

#### Confidentiality

All participant information, including laboratory test results, is securely stored. Blood samples and study records are identified using a unique base ID. Documents containing personal identifying information are stored separately in a secure location. In addition to the study team, only authorized personnel have access to participant records for study monitoring purposes.

## Results

This study was initiated on September 5, 2023. Total enrollment of 1510 participants (n=755, 50% in each group) has been completed by screening 1644 participants. As of August 14, 2025, a total of 1137 participants had successfully completed the study, 280 (24.62%) are continuing, and 93 (8.18%) have dropped out (n=48, 51.61% in the intervention group and n=45, 48.39% in the control group).

## Discussion

### Expected Findings

In Ayurveda, disease management focuses on understanding the pathogenesis of the condition. SCA can be explained in terms of *raktavaha srotas dushti*, which refers to the imbalance or dysfunction of the hematopoietic system. In some studies, it has been correlated with *sannipataja pandu roga* (advanced or multifactorial condition of anemia leading to complex blood disorders with systemic symptoms) [21,22]. The *yakrita* (liver), *pleeha* (spleen), and *amashaya* (stomach) are the sites involved in the transformation of the *rasa* (essence of the digestive food elements) into *rakta* (blood) with the help of *ranjaka pitta* [23] (intrinsic factor responsible for erythropoiesis), a predominant factor for the formation of *rakta* [24]. The *beejadushti* [25] (genetic mutation) may cause altered development and functioning of the *raktavaha srotas* as well as *ranjaka pitta*, thereby hampering the proper formation of *rakta dhatu* (vitiation of blood). The depletion is accompanied by imbalances in other *dhatus* (body tissues), aggravating *vata dosha* (body humor) [26]. The clinical features of *raktavha srotas dushti* comprise *jeerna jwara* (chronic fever), *yakritodara* (hepatomegaly), and *plihodara* (splenomegaly), among others [27]. The pathology in *raktavaha srotas* can lead to several diseases, including *raktapitta* [28] (bleeding disorder), which may be associated with SCD. It subsequently results in complications such as *raktakshaya* (deficiency of blood), identified through *sirashaithilya* (deformity of blood vessels) and manifestations in the appearance of the skin [29].

In SCA, the pathogenesis is caused by a hemoglobinopathy that leads to the sickling of RBCs. Due to increased oxidative stress and production of reactive oxygen species [30], it further leads to endothelial dysfunction, cell adhesion, vaso-occlusion, and hemolysis. The resultant tissue ischemia and infarction are responsible for the acute systemic painful vaso-occlusive crisis [31] and anemia as the extent of compensatory increased RBC production is insufficient to balance the increased rate of destruction [32]. Consequently, it affects systemic organs such as the liver, spleen, kidneys, and lungs and causes neurological, cardiovascular, renal, and urological complications [33].

There are various drugs in Ayurveda for the management of *raktadushti* (vitiation of blood), among which *dadimadi ghrita* was selected in this study due to its *rakta vardhana* (increases blood) and *balavardhaka* (increases strength) action [34]. The formulation is based on ghee, which itself has properties such as *brumhana* (nourishing or promoting growth) and *pushtikara* (providing strength or vitality), aiding in pacifying *vata dosha* [35]. It contains potent ingredients such as *chitraka* (*Plumbago zeylanica*), *dhanyaka* (*Coriandrum sativum*), *shunthi* (*Zingiber officinale*), and *pippali* (*Piper longum*), which are known for their *deepana* and *pachana* properties [36,37]. These ingredients support the elimination of *ama* (toxic metabolic waste), thereby aiding in detoxification through their *pachana* properties. By enhancing digestive efficiency, the formulation promotes better assimilation of nutrients and supports *dhatuposhana*—the nourishment of bodily tissues—while helping regulate imbalanced *doshas* and improve overall metabolic function [38]. Furthermore, the antioxidant properties of *dadima* (*Punica granatum*), *dhanyaka* (*C sativum*), and *shunthi* (*Z officinale*) may help mitigate oxidative stress, which is especially relevant in conditions such as SCA, potentially aiding in the prevention and management of related complications [39-41].

The other interventional drug, Ayush-RP, is a coded drug developed by the Central Council for Research in Ayurvedic Sciences. According to a pilot study conducted previously on different tribal populations residing in Maharashtra, Ayush-RP showed significant improvements in pain crisis episodes and quality of life for participants diagnosed with SCA [13]. The ingredients have *raktaprasadan karma* [42], an action that improves blood quality by restoring balance to the dysregulated *pitta dosha*, eliminating vitiated factors, and modulating the components of the blood. This may help alleviate *raktadushti* and further facilitate the free circulation of blood. The action of the constituents, such as *lekhana karma* (scrapping of unwanted tissues), helps in the elimination of *sanga-srotodushti* (obstruction in minute channels of the tissues). Consequently, it may reduce vaso-occlusion and further mitigate painful crises. Additionally, the *shonitasthapana* action may regulate hemolysis and enhance blood volume. *Ruksha, sheeta guna*, *tikta-kashaya rasa*, *madhura vipaka*, and *sheeta veerya* in the formulation pacify *tridosha* (all 3 body humors) and *raktadushti*, and its ingredients are specifically mentioned in Ayurveda texts for managing *rakta vikara* (blood disorders), particularly *raktapitta*. In addition, the components support *dhatuposhana*, which may improve the overall strength and vitality of the body. The formulation comprises nanoactive particles that increase the bioavailability of the compound, assisting in better absorption and assimilation. The ingredients of the formulation have antipyretic, analgesic, anti-inflammatory, immunomodulatory, antioxidant, and hepatoprotective properties and are considered to elevate hemoglobin levels as per pharmacognostical, preclinical, and clinical studies.

Hydroxyurea is the primary drug in the management of SCA and is currently the most effective disease-modifying therapy for individuals with SCA, reducing the production of rapidly dividing cells, including those involved in the inflammatory processes in SCD. It increases the production of fetal hemoglobin (HbF), which helps reduce sickling of RBCs and the frequency of painful episodes. It also increases overall hemoglobin levels, improving oxygen delivery and reducing the severity of symptoms [43]. In SCA, increased erythropoiesis often occurs as the body attempts to compensate for the rapid turnover of sickled RBCs. This heightened demand for new RBCs results in a greater need for folic acid, a crucial vitamin involved in DNA synthesis and cell division. For this reason, folic acid is prophylactically recommended in standard care [44].

Overall, these interventions, when applied in an integrated approach, are expected to mitigate vaso-occlusion, reduce oxidative stress and inflammation, limit the extent of hemolysis and organ damage, and decrease the frequency of pain crises and the need for blood transfusions. Collectively, these effects contribute to an improved quality of life in individuals living with SCD.

### Strengths and Limitations of This Study

A key strength of this study is its rigorous design as a randomized active-controlled trial, which enhances the reliability of the findings. Additionally, the innovative integration of standard care with Ayurvedic interventions offers a holistic approach that may provide therapeutic benefits in the management of SCA. However, this integrated approach presents challenges in isolating the specific effects of the Ayurvedic component, making it difficult to determine its independent contribution to the overall outcome.

### Future Scope

A future randomized controlled trial could be designed to compare the efficacy of Ayurvedic interventions versus the standard therapeutic care in the management of SCA, facilitating a precise evaluation of the effectiveness of each approach.

### Conclusions

SCA, being a hereditary condition, is currently devoid of a definitive treatment through either conventional or traditional methods and is instead prevented and managed symptomatically.

This study may contribute to establishing the efficacy of Ayurvedic interventions as an integrated approach to managing SCA, aiming to reduce the frequency of pain crises and enhance patients’ overall quality of life.

## Abbreviations

AE: adverse event
CRF: case report form
DMIHER: Datta Meghe Institute of Higher Education and Research
RBC: red blood cell
SCA: sickle cell anemia
SCD: sickle cell disease
